# Adverse Effects of Busulfan Plus Cyclophosphamide versus Busulfan Plus Fludarabine as Conditioning Regimens for Allogeneic Bone Marrow Transplantation

**DOI:** 10.31557/APJCP.2021.22.5.1639

**Published:** 2021-05

**Authors:** Mahshid Mehdizadeh, Sayeh Parkhideh, Sina Salari, Abbas Hajfathali, Hamid Rezvani, Maryam Mabani

**Affiliations:** 1 *Department of Hematopoietic Stem Cell Transplantation, Taleghani Hospital, Shahid Beheshti University of Medical Seience, Tehran, Iran. *; 2 *Department of Hematology and Oncology, Taleghani Hospital, Shahid Beheshti University of Medical Seience, Tehran, Iran. *

**Keywords:** Conditioning regimen, safety, allogeneic BMT

## Abstract

**Background:**

The side effects of conditioning regimens on the success rate of allogeneic transplantation around the world have been challenging. In this study, we aimed to investigate the side effect of Bu/Cy and Bu/Flu regimens on our patients who underwent allogeneic bone marrow transplantation.

**Methods:**

We analyzed 180 patients receiving bone marrow transplantation in Taleghani Hospital, in Tehran, Iran between April 2016 and December 2019. Patients in group A received a combination of intravenous busulfan 0.8 mg/kg QID over two hours for 4 consecutive days (12.8 mg/kg in total)(Savani et al., 2006) and cyclophosphamide 60 mg/kg per day for two consecutive days. Patients in group B received busulfan the same as the first group in combination with fludarabine equal to 40 mg/m² per day. Patients were followed up at regular intervals up to two years after transplantation.

**Result:**

Various items were evaluated for patients, including cardiopulmonary function, psychological disorders, GVHD, and endocrine disorders such as hypothyroidism, fertility, or gonad dysfunction. Primary hypothyroidism developed in 13.3% and 11.1% of the Bu/Cy and Bu/Flu groups, respectively (p=0.230). None of the patients in either group experienced infertility or gonad dysfunction. In group A versus group B, pulmonary diseases were detected in 4.4% versus 6.6% of BMT recipients, respectively (p = 0.223). In both groups, mitral and tricuspid regurgitation were observed in patients (8.9% vs. 11.1%; p = 0.189). Incidence of Psychological disorders was no significant difference between the two groups. 32.2% of group A versus 34.45% of group B had skin and liver GVHD, respectively (p = 0.235).

**Conclusion:**

The therapeutic-related adverse effects of the two conditioning regimens in patients who underwent allogeneic bone marrow transplant were almost similar. To improve quality of life and overall survival among BMT patients, careful evaluation of treatment-related complications should be part of the regular follow-up of them.

## Introduction

Bone marrow and blood stem cell transplants (BMT) actually replace a person’s abnormal stem cells with those of a healthy person (a donor) so that the patient can receive high doses of chemotherapy or radiation therapy. This method allows the recipient to access new stem cells that are functioning properly (Peccatori and Ciceri, 2010; Niederwieser et al., 2016). This treatment method is used to cure many different malignant and benign hematological and non-hematological diseases. The survival of many patients after bone marrow transplantation is increased and there is a significant improvement in their quality of life (Majhail et al., 2015).

In recent years, bone marrow transplants have been carried out in two ways: autologous and allogeneic transplantation. In an allogeneic transplant, the patients receive tissue from someone other than themselves or an identical twin, such as a brother, sister, parent, or unrelated person (Arcese et al., 2011). In this method, the transplant donor and the patient must match exactly in terms of the major histocompatibility complex (MHC). The more similar the human leukocyte antigen (HLA) components are, the lower is the risk of graft-versus-host transplant (GVHD) and rejection of the graft. After selecting the tissue donor and ensuring the health of the patient and the donor, the main stages of the transplant begin (Passweg et al., 2017).

After preparing the transplant tissue, the patient is given high doses of chemotherapy drugs with or without irradiation to destroy the remaining bone marrow (Holter-Chakrabarty et al., 2015). Finally, the healthy bone marrow is warmed and injected into the patient through a vein to replace the destroyed bone marrow. Transplanted cells in the bone marrow produce new white blood cells (WBC), red blood cells (RBC), and platelets. Infection and bleeding, nausea/vomiting, fatigue, anorexia, mouth ulcers, hair loss, and skin reactions are some of the side effects of BMT (Shanklin et al., 2018).

This treatment method, like any other methods, affects various organ systems of the body and leads to acute and chronic complications. The rate and severity of post-transplant complications on the neuro-hormonal systems are affected by pre-transplant conditioning regimens (Inamoto and Lee, 2017). Transplant preparative or conditioning regimens include total body irradiation (TBI), often in combination with chemotherapy or chemotherapy alone (Paix et al., 2018). Such regimens are determined based on factors such as the patient’s age at the time of transplantation, gender, the stage of the underlying disease at the time of transplantation, pubertal status, primary disease, type of transplant, history of receiving chemotherapy before BMT, and GVHD (Metheny and de Lima, 2019). 

One transplant conditioning regimen is based on the combination of intravenous busulfan plus fludarabine (Bu/Flu). Previous studies (Jethava et al., 2017; Xu et al., 2017) have shown that the Bu/Flu regimen reduces relapse-related mortality and injection time compared with the regimen of busulfan plus cyclophosphamide (Bu/Cy) (Jethava et al., 2017; Xu et al., 2017). Some studies, however, obtained conflicting results. For example, one study (Rambaldi et al., 2015) concluded that the Bu/Flu regimen was not a suitable alternative to the Bu/Cy regimen because of the increased recurrence rate; another study (Saraceni et al., 2018) on young patients with acute myeloid leukemia compared the Bu/Flu and Bu/Cy regimens and found that the Bu/Flu regimen was less toxic than the Bu/Cy regimen but had similar anti-leukemic activity (Rambaldi et al., 2015; Saraceni et al., 2018).

The current study was performed to address the lack of a comprehensive evaluation and comparison of the results and side effects of Bu/Cy and Bu/Flu regimens in patients who have undergone allogeneic transplant in Iran and the existence of conflicting studies around the world. 

## Materials and Methods

*Subjects*

**Figure 1:**
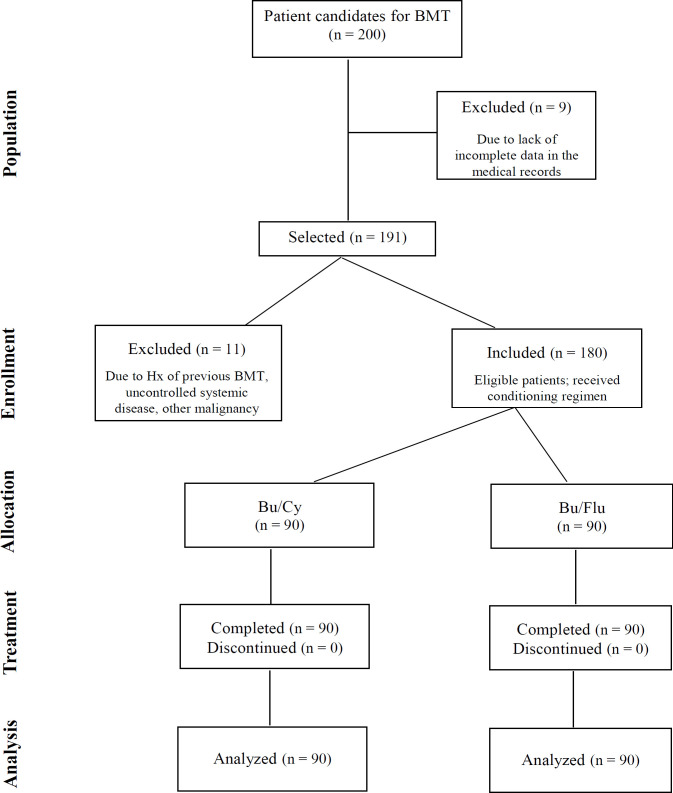
Flow Diagram of Patient Selection

This retrospective cohort was conducted on patients who referred to the bone marrow transplantation unit in Taleghani Hospital, in Tehran, Iran, from April 2016 to December 2019. Inclusion criteria comprised being an adult (18-70 years) and a candidate for allogeneic bone marrow transplantation. Exclusion criteria were having a history of previous BMT, other primary malignancies, active infection, pregnancy, or uncontrolled systemic diseases (including severe cardiovascular, pulmonary, renal and/or hepatic failure) and refusal to participate in the study (Figure 1).

*Data Collection*

This retrospective cohort study was approved by the Ethics Committee of Shahid Beheshti University of Medical Sciences. All patient data was kept confidential. The medical records of eligible patients who were candidates for allogeneic BMT were studied. Initially, the demographic characteristics of patients, such as age and sex were recorded in the data collection form. Then, the patients were randomly divided into two groups: 1) Intervention group A: Patients received a combination of intravenous busulfan 0.8 mg/kg QID over two hours for 4 consecutive days (16 doses from day -9 to day -6; in total, 12.8 mg/kg) and cyclophosphamide 60 mg/kg per day for two consecutive days (on days -4 and -3; in total, 120 mg/kg); 2) Intervention group B: Patients received busulfan 0.8 mg/kg QID over two hours for 4 consecutive days (from days -6 to -3) in combination with fludarabine equal to 40 mg/m² per day for 4 consecutive days (from days -6 to -3) with a total dose of 160 mg/m². All patients received the same dose of busulfan, and no dose reduction or discontinuation was allowed. On day 0, patients received bone marrow cells in their transplant. To prevent GVHD, patients received intravenous cyclosporine A 1.5 mg/kg bid on day -1 before transplantation (to reach the target level of 200 ng/mL or more), intravenous methotrexate 15 mg/kg m² on day 1, and subsequently 10 mg/m² on days 3, 6, and 11. Patients in both intervention groups who received stem cells from unrelated donors were treated with intravenous anti-thymocyte immunoglobulin equal to 0.5 mg/kg on day -3, and 0.2 mg/kg on day -2. In cases where there was an antigen or allele disorder between the donor and the recipient, the final dose of anti-thymocyte immunoglobulin was increased up to 7.5 mg/kg.

After BMT, patients were followed up, and the main data of results and side effects on days 30, 60, 100, and 180 and then in the first and second year after BMT were extracted from the medical records. The ratios of donor and recipient bone marrow cells, blood cells, and T-cells were concurrently evaluated. Patients were assessed weekly in terms of symptoms related to acute GVHD with organ involvement in the first three months after BMT. Cases were followed to evaluate chronic GVHD at each examination.

*Statistical analysis*

All statistical analyses were performed using IBM SPSS statistics 24.0 software (IBM Corporation, Armonk, NY, USA). The frequency of the studied variables was reported as mean and standard deviation (Sun et al.) and percentage. To compare the side effects of two conditioning regimens, the chi-square test was used. In all analysis, a p-value under 0.05 was considered statistically significant.

## Results

A total of 180 patients who had undergone BMT were enrolled in the study. In the Bu/Cy group (n = 90), 54 cases (60%) had AML and 36 cases (40%) had ALL; in the Bu/Flu group (n = 90), 49 persons (54.4%) had AML and 41 (45.5%) had ALL (p = 0.256). In the Bu/Cy group, 48 cases (53.3%) were males and 42 cases (46.7%) females, and in the Bu/Flu group, 43 cases (47.7%) were males and 47 cases (52.3%) females (p = 0.103). In the Bu/Cy group versus the Bu/Flu group, patients received tissue from HLA-identical siblings (87.7% vs. 91.1%), other related (10.0% vs. 7.8%), and unrelated donors (2.2% vs. 1.1%) (p = 0.396).

Dry eye was observed in 31.1% (n=28) of patients in the Bu/Cy group, of which 11.1% (n=10) developed cataract, and 33.3% (n=30) of patients in the Bu/Flu group, of which 12.2% (n=11) developed cataract (p = 0.480). In the Bu/Cy group, 13.3% of patients (n=12) and in the Bu/Flu group, 11.1% of patients (n=10) developed primary hypothyroidism (p = 0.230). None of the BMT survivors in either group experienced infertility or gonad dysfunction. In the Bu/Cy and Bu/Flu groups, respectively, 12 of 42 female patients and 14 of 47 female patients did not take LH and FSH tests. According to LH and FSH levels, 14.3% and 9.5% of female patients in the Bu/Cy group and 14.9% and 8.5% of female patients in the Bu/Flu group, respectively, had ovarian failure (p = 0.555). Among the male transplant patients, 5 of 48 in the Bu/Cy group and 4 of 43 in the Bu/Flu group did not take hormonal tests. Testosterone levels were lower than normal in 38 men (79.1%) in the Bu/Cy group and in 36 men (83.7%) in the Bu/Flu group based on their FSH and LH levels. In the Bu/Cy group, 18 men (37.5%) had secondary hypogonadism and 26 men (54.1%) had primary gonad dysfunction In the Bu/Flu group, these numbers were 15 (43.9%) and 22 (51.1%), respectively (p = 0.318, p = 0.228, respectively).

In the Bu/Cy group versus the Bu/Flu group, obstructive and restrictive pulmonary diseases were detected in 13.3% and 4.4% versus 15.54% and 6.6% of BMT recipients, respectively (p = 0.306, p = 0.223, respectively). In both groups, mitral and tricuspid regurgitation (MR/TR) were found in patients (n=8, 8.9% in the Bu/Cy group versus n=10, 11.1% in the Bu/Flu group) (p = 0.189). Pericardial effusion occurred in 1 patient (1.11%) in the Bu/Cy group and in 2 cases (2.2%) in the Bu/Flu group (p = 0.760). Moreover, right bundle branch block (RBBB) was observed in 1 patient (1.11%) in the Bu/Cy group and in no patient in the Bu/Flu group (p = 0.899).

Transplant-related psychological distress was very common among recipients in both groups. In the Bu/Cy group versus the Bu/Flu group, psychological disorders were observed in 38.8% vs. 39.9% of BMT survivors (p = 0.902), of whom 84.2% vs. 83.3% were diagnosed with depression and others with anxiety disorders and insomnia. One patient (1.11%) presented with convulsion in the Bu/Flu group. One patient (1.11%) was affected by astrocytoma in the Bu/Cy group. Moreover, 29 cases (32.2%) in the Bu/Cy group and 31 cases (34.4) in the Bu/Flu group experienced chronic and non-pulmonary GVHD, including skin and liver GVHD (p = 0.235).

**Table 1:** The Clinical Characteristics of Leukemia Patients Underwent BMT

	No. of patients	Type of leukemia (%)		Gender (%)		BMT (%)		
		AML	ALL	Female	Male	HLA-identical siblings	Related	Unrelated
Bu/Cy	90	54	36	42	48	87.7	10	2.2
Bu/Flu	90	49	41	47	43	91.1	7.8	1.1

**Table 2:** List of Post-BMT Side Effects according to the Chemotherapeutic Regimen

Post-BMT side effects	Bu/Cy	Bu/Flu
Dry eye	31.1%	33.3%
Primary hypothyroidism	13.3%	11.1%
Obstructive pulmonary diseases	13.3%	15.54%
Restrictive pulmonary diseases	4.4%	6.6%
Pericardial effusion	1.1%	2.2%
Right bundle branch block (RBBB)	1.1%	0.0%
Transplant-related psychological distress	38.8%	39.9%
Convulsion	0.0%	1.1%
Astrocytoma	1.1%	0.0%
Chronic GVHD	32.2%	34.3%

## Discussion

No significant differences were observed in the 2-year follow-up between the two study groups in terms of post-transplant complications in patients who had undergone allogeneic BMT. Dry eye was the most frequent ophthalmic complication after BMT in both groups. A significant percentage of patients in both groups had hormonal disorders, especially sex hormone disorders. Mitral and tricuspid regurgitation (MR/TR) were the most common cardiac complications among patients in both groups. Pulmonary diseases were observed in less than 20% of patients in both groups. The majority of BMT cases in both groups had psychological disorders, especially depression. Approximately one third of the BMT patients in both groups developed chronic GVHD.

BMT is a curative treatment for a variety of hematological diseases. The safety of HCT has improved over the decades. Indications for BMT have expanded to older patients, and almost all patients are able to find proper allogeneic donors by the growing use of cord blood and haplo-identical transplantation (Gooley et al., 2010; Sorror et al., 2011). These current conditions have contributed to a growing number of BMT survivors, estimated to be half a million worldwide. Patients who are disease-free at two or five years after BMT have a greater than 80% subsequent 10-year survival rate, but many studies have shown that BMT survivors suffer from significant late effects that adversely affect morbidity, mortality, working status, and quality of life (Martin et al., 2010; Wingard et al., 2011). A prospective observational study (Sun et al., 2010) of 1022 survivors who underwent HCT between 1974 and 1998 showed that 66% of the survivors had at least one chronic condition and 18% had severe or life-threatening conditions (Sun et al., 2010). A retrospective study (Khera et al., 2012) of 1087 contemporary survivors also showed that the cumulative incidence of any non-malignant late effect at five years after BMT was 45% among autologous and 79% among allogeneic recipients, and 2.5% of autologous and 26% of allogeneic recipients had three or more late effects (Khera et al., 2012). It has been reported (Martin et al., 2010; Wingard et al., 2011) life expectancy after BMT among 5-year survivors remained 30% lower compared with the general population, regardless of their current ages and amount of time passed. The leading causes of deaths in 5-year survivors included secondary malignancies (27%) and recurrent disease (14%), followed by infections (12%), GVHD (11%), cardiovascular diseases (11%), and respiratory diseases (7%) (Martin et al., 2010; Wingard et al., 2011).

The conditioning regimens for BMT are also associated with long-term complications. In a retrospective analysis (Ringden et al., 2014) of 4269 patients with AML, MDS, and lymphoma who underwent BMT, there was a higher risk for cancers of oral sites (lip, tonsil, oropharynx), bone, soft tissue, vulva, and melanoma, with age (> 50 years) being the only independent risk factor for solid cancers in AML and MDS patients (Ringdén et al., 2014). In the present study, however, only one patient in the Bu/Cy group developed a solid tumor (astrocytoma). Similarly, a retrospective analysis of 931 patients with a cyclophsphomide-based regimen (n=257), reduced intensity conditioning (RIC) (n=449), or reduced toxicity conditioning (RTC) (n=225) found the incidence rates of secondary malignancies to be 1.7%, 7.4%, and 5.7% post-BMT, respectively. On multivariate analysis, FLU-based conditioning, moderate-severe GVHD, and chronic myeloproliferative or non-malignant disease were identified as risk factors for secondary malignancy; in the present study, almost one-third of cases in both conditioning groups experienced GVHD. The risk of secondary malignancies is not reduced in the period of conditioning; the risk is lifelong (Shimoni et al., 2013; Shimoni, 2014). Hypogonadism and infertility remain important clinical problems for patients after receiving preparative regimens for BMT. All patients of reproductive age should be counseled on this important complication of transplantation (Rovó et al., 2006; Savani et al., 2006).

A large number of various conditioning regimens have been tested in patients with hematological disorders such as AML or ALL. When an allogeneic BMT is indicated, possibly in all intermediate-/high-risk patients, the conditioning regimen can be tailored according to the age and comorbidities of the patient. Young patients under the age of 45 will most likely benefit from a conditioning regimen such as Bu/Cy. Between 45 and 65 years of age, Bu/Cy may be toxic, and Bu/Flu would be the first choice. In patients above the age of 65, a RIC regimen is probably the best option and could be tested up to the age of 70 and over, possibly, but not necessarily, preceded by a short course of chemotherapy. In these elderly patients, due to dismal current results of induction chemotherapy, upfront transplantation may be worth a clinical trial.

In conclusion, the results of this study showed that the side effects of two conditioning regimens in patients who had undergone allogeneic bone marrow transplant were relatively similar. To improve the quality of life and overall survival in BMT patients, careful evaluation of treatment-related complications should be part of the regular patient follow-up. Diagnostic and therapeutic interventions must also be taken into account to prevent, diagnose early, and treat the side effects of BMT. Survivors should be screened for evidence of hormonal disturbances at their periodic health examinations. There should also be regular periodic ocular, cardiovascular, pulmonary, and mental status examinations. Because of the high risk of infertility in BMT survivors, it is recommended to store male’s sperm and female’s ovule prior to BMT to preserve fertility in adult patients. However, these results should be confirmed by larger studies in the future.

## Author Contribution Statement

M Mehdizadeh conceived the idea and wrote the manuscript. SP wrote the manuscript and prepared the table. SS participated in data gathering and writing of manuscript. AH revised the manuscript. HR provided figures and wrote the manuscript. M Mabani supervised the project and revised the manuscript.

## Fundings

The authors thank Shahid Beheshti University of Medical Sciences for their financial support of this study.

This research was approved by the stem cell transplant specialists at Taleghani Hospital.
